# Hematological Adverse Events with Tyrosine Kinase Inhibitors for Chronic Myeloid Leukemia: A Systematic Review with Meta-Analysis

**DOI:** 10.3390/cancers15174354

**Published:** 2023-08-31

**Authors:** Olivia Kronick, Xinyu Chen, Nidhi Mehra, Armon Varmeziar, Rachel Fisher, David Kartchner, Vamsi Kota, Cassie S. Mitchell

**Affiliations:** 1 Laboratory for Pathology Dynamics, Department of Biomedical Engineering, Georgia Institute of Technology and Emory University School of Medicine, Atlanta, GA 30332, USA; 2 Department of Medicine, Hematology and Oncology, Georgia Cancer Center at Augusta University, Augusta, GA 30912, USA; vkota@augusta.edu; 3 The Machine Learning Center at Georgia Tech, Georgia Institute of Technology, Atlanta, GA 30332, USA

**Keywords:** chronic myeloid leukemia, tyrosine kinase inhibitor, personalized medicine, Philadelphia chromosome

## Abstract

**Simple Summary:**

Tyrosine kinase inhibitors (TKIs) are the main class of drugs used to treat chronic myeloid leukemia. Because most CML patients must remain on TKIs indefinitely, it is important to understand and monitor adverse events (AEs). Changes to blood cells, or hematological adverse events, are often attributed to CML, itself. However, once the disease is stabilized, changes in red blood cells, white blood cells, or platelets, may be due to the TKI therapy. This study reports the frequency of hematological AEs in CML patients treated with TKIs, which has implications in TKI selection and patient monitoring.

**Abstract:**

Chronic myeloid leukemia (CML) is treated with tyrosine kinase inhibitors (TKI) that target the pathological BCR-ABL1 fusion oncogene. The objective of this statistical meta-analysis was to assess the prevalence of other hematological adverse events (AEs) that occur during or after predominantly first-line treatment with TKIs. Data from seventy peer-reviewed, published studies were included in the analysis. Hematological AEs were assessed as a function of TKI drug type (dasatinib, imatinib, bosutinib, nilotinib) and CML phase (chronic, accelerated, blast). AE prevalence aggregated across all severities and phases was significantly different between each TKI (*p* < 0.05) for anemia—dasatinib (54.5%), bosutinib (44.0%), imatinib (32.8%), nilotinib (11.2%); neutropenia—dasatinib (51.2%), imatinib (29.8%), bosutinib (14.1%), nilotinib (14.1%); thrombocytopenia—dasatinib (62.2%), imatinib (30.4%), bosutinib (35.3%), nilotinib (22.3%). AE prevalence aggregated across all severities and TKIs was significantly (*p* < 0.05) different between CML phases for anemia—chronic (28.4%), accelerated (66.9%), blast (55.8%); neutropenia—chronic (26.7%), accelerated (63.8%), blast (36.4%); thrombocytopenia—chronic (33.3%), accelerated (65.6%), blast (37.9%). An odds ratio (OR) with 95% confidence interval was used to compare hematological AE prevalence of each TKI compared to the most common first-line TKI therapy, imatinib. For anemia, dasatinib OR = 1.65, [1.51, 1.83]; bosutinib OR = 1.34, [1.16, 1.54]; nilotinib OR = 0.34, [0.30, 0.39]. For neutropenia, dasatinib OR = 1.72, [1.53, 1.92]; bosutinib OR = 0.47, [0.38, 0.58]; nilotinib OR = 0.47, [0.42, 0.54]. For thrombocytopenia, dasatinib OR = 2.04, [1.82, 2.30]; bosutinib OR = 1.16, [0.97, 1.39]; nilotinib OR = 0.73, [0.65, 0.82]. Nilotinib had the greatest fraction of severe (grade 3/4) hematological AEs (30%). In conclusion, the overall prevalence of hematological AEs by TKI type was: dasatinib > bosutinib > imatinib > nilotinib. Study limitations include inability to normalize for dosage and treatment duration.

## 1. Introduction

Encompassing about 15% of all newly diagnosed cases of adulthood leukemia, chronic myeloid leukemia (CML) is a disease afflicting early myeloid cells of the bone marrow [1]. CML is characterized by the presence of what is known as a Philadelphia chromosome, which results from the fusion of the breakpoint cluster region (BCR) gene found on chromosome 22q11.2 with the Ableson gene (ABL1) from chromosome 9q34. This genetic translocation results in a BCR-ABL fusion and a resulting fused BCR-ABL oncoprotein. The oncogene then produces the BCR-ABL protein, a type of tyrosine kinase, which causes CML blood cells to grow and divide out of control. Multiple studies have summarized the molecular biology of BCR-ABL leukemia e.g., [2,3]. There are three stages of CML: the chronic phase, the accelerated phase, and the blast phase. Classification of a patient by stage primarily depends on the number of blasts in the blood or bone marrow. As of 2022, chronic myeloid leukemia has an incidence of 1–2 cases per 100,000 adults. Chronic myeloid leukemia prevalence has been increasing, with over 150,000 total cases in 2022 with expected growth in the coming decades [1].

CML effects the myeloid cell lineage in the bone marrow, which means there is a potential for abnormalities in the white blood cells (WBC), red blood cells (RBC), and platelets. However, the primary hematological initial finding for active CML is absolute leukocytosis, which is typically defined as an elevated absolute WBC greater than 12,000 leukocytes per microliter [4]. At initial diagnosis, CML patients typically have an absolute WBC count greater than 25,000 leukocytes per microliter with a median of 100,000 leukocytes per microliter [5,6]. Beyond primary leukocytosis, abnormalities at the time of initial CML diagnosis may include anemia (low absolute RBC count), thrombocytosis (high absolute platelet count), or thrombocytopenia (low absolute platelet count). Prior works have estimated that 34.8% of newly diagnosed chronic phase adult CML patients present with co-existing anemia [7], whereas 81% of newly diagnosed pediatric CML patients experience anemia at the time of diagnosis [8].

At the turn of the century, the development of a modern small-molecule BCR-ABL tyrosine-kinase inhibitor (TKI) imatinib mesylate profoundly improved CML outcomes [9]. A 10-year follow-up study of imatinib showed a complete cytogenetic response in 83% of patients [10]. A complete cytogenetic response is defined as detection of 0% Ph+ metaphases and indicates improved survival and life expectancy [11]. Following a rise in BCR-ABL resistance to imatinib, second generation TKIs were developed, including nilotinib, bosutinib, and dasatinib [9]. Eventually, third line TKIs to were approved for CML [12], such as ponatinib and radotinib. The success of TKIs saw a reduction in annual morbidity rates from 20% in 2000 to a 1–2% morbidity rate in 2021 [1]. Because of this substantial increase in survival rates, CML treatment has shifted toward that of a “chronic” condition, with CML patients continuing TKI therapy indefinitely.

While TKIs dramatically increased survival, their effects are not benign. Both acute and chronic adverse events (AEs) have been reported in association with the usage of TKIs as frontline therapy for CML. For example, bosutinib was found to have the highest prevalence of gastrointestinal AEs, with 79.2% of patients experiencing diarrhea and 42.4% experiencing nausea [13]. Nilotinib was issued a black-box warning by the United States Food and Drug Administration (FDA) in 2020 in response to heart concerns with Q-T prolongation [14]. Pleural effusion (PE) is a notable AE associated with dasatinib usage, where PE was 33 times more frequent with dasatinib compared to imatinib [15,16]. Ponatinib has been associated with a serious risk of arterial occlusion and hepatotoxicity in some patients, which initially resulted in a removal of the drug from market before its re-release [17]. High incidence of renal dysfunction in imatinib users has been reported in clinical trials, in addition to severe cases of liver failure [18]. Fatigue and thrombocytopenia are common AEs attributed to asciminib [19] and radotinib, where it occurred in 24% of patients [20]. Ruxolitinib [21] and tipifarnib [22] have also shown a strong propensity for fatigue and gastrointestinal AEs in follow-up studies.

CML therapy has shifted towards long-term disease management and maintaining a deep molecular response according to the BCR-ABL polymerase chain reaction (PCR) test on peripheral blood. PCR measures the number of BCR-ABL transcript copies in the peripheral blood levels. A deep molecular response corresponds to 0.01% on the International Scale, which means that less than or equal to 1:10,000 measured transcripts possess the mutation (e.g., a 4-log reduction compared to the average number of BCR-ABLE copies at initial diagnosis) [23].

CML treatment with TKIs is considered a life-long therapy. In recent years, treatment-free remission (TFR) has emerged as a goal for CML patients who achieve a deep molecular response to TKI treatment. However, TFR is still only achievable in a subset of patients, with studies citing as many as 44–56% of patients experience a molecular relapse within four months of treatment discontinuation [23,24,25,26]. While TFR remains a goal for continued progress in TKI therapy, presently a majority of CML patients must remain on lifelong TKI therapy. Improved assessments for long-term AEs associated with TKI therapy remains a key frontier of TKI treatment research.

A previous cross-domain text-mining study utilized machine learning patterns to identify possible under-reported AE relationships in CML patients on long-term TKI therapy. Predicted results showed hematological AEs were the top-ranking AEs associated with TKI usage in CML patients [13]. Once a CML patient obtains a complete hematological response, and especially a deep molecular response, hematological abnormalities should no longer be attributed to the CML pathology, itself. Later occurring or re-occurring hematological AEs while on TKI therapy could be attributed to the TKI, itself, and/or other co-morbidities. Hematological AEs may exacerbate quality of life by causing fatigue (due to anemia); susceptibility to infection (due to neutropenia); or abnormalities in clotting (due to thrombocytopenia or thrombocytosis).

Therefore, the goal of this study was to determine the prevalence of hematological AEs at various stages of CML treatment and to determine the hematological AE risk profile for each of the most common TKIs. As noted above, hematological AEs have been previously examined in published individual clinical trials or cohort studies for each TKI. However, statistical meta-analysis is necessary to combine multiple cohorts and estimate the aggregated effect size of hematological AEs for each main TKI. The ability to look at hematological AE frequency across cohorts lessens study-specific biases and draws additional attention to the need for appropriate TKI selection and dose titration. To this end, we perform a systematic review and meta-analysis to assess anemia, thrombocytopenia, and neutropenia (absolute low WBC counts) in CML patients taking TKIs. Present findings can be used to direct future TKI research areas in an effort to improve TKI design and lessen AE occurrence. Additionally, findings can be used to make connections between AEs and the causal TKI treatment. Personalizing TKI treatment for CML patients based on medical history has implications for minimizing AE incidence and severity and increasing patient compliance to the treatment regimen.

## 2. Materials and Methods

The objective was to assess the prevalence and correlation of hematological conditions or AEs in CML patients on TKI treatment. Data sources were assessed utilizing searches on PubMed.gov and ClinicalTrials.gov to discover relevant peer-reviewed data sources for performing a cross-cohort meta-analysis. The prevalence of each hematological condition was examined and then compared across specific TKI treatments and different CML disease stages. Ultimately, sufficient data was available to assess the prevalence of anemia, neutropenia, or thrombocytopenia while being treated with imatinib, dasatinib, nilotinib, or bosutinib for the chronic phase, accelerated phase, or blast phase of CML.

### 2.1. Protocol and Registration

Methods and reporting used in this study conforms to the Reporting Items for Systematic Reviews and Meta-Analyses (PRISMA) guidelines and is registered with the International Prospective Register of Systematic Reviews, CRD42023405802.

### 2.2. Search Strategy

The following were performed on PubMed.gov and ClinicalTrials.gov. Searches were completed through end of the year 2021. The key words used to search were as follows:

- (“chronic myeloid leukemia” OR “chronic myelogenous leukemia” OR “CML) AND (“imatinib” OR “dasatinib” OR “nilotinib” OR “bosutinib” OR “ponatinib” OR “asciminib” OR “radotinib” OR “ruxolitinib” OR “tipifarnib”)
- (“chronic myeloid leukemia” OR “chronic myelogenous leukemia” OR “CML) AND (“aplastic anemia” OR “anemia” OR “neutropenia” OR “thrombocytopenia” OR “myelosuppression” OR “pancytopenia”).

**Figure 1:**
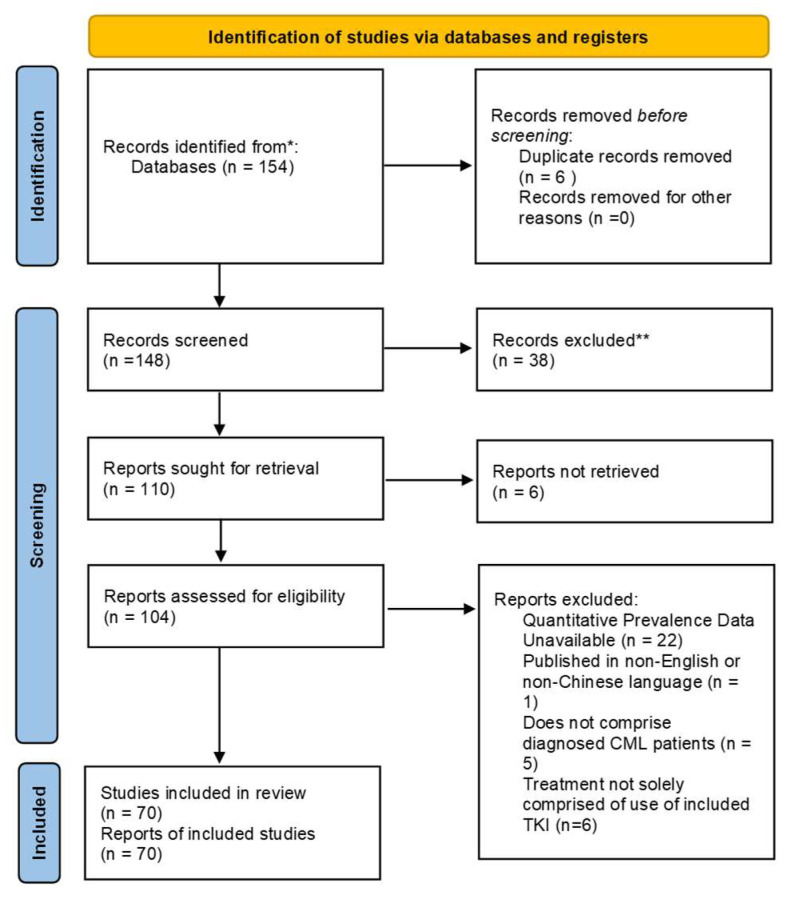
PRISMA diagram for study inclusion. The * indicates initial search parameters and ** indicates screened review.

Two unblinded reviewers screened articles yielded by searches against specified inclusion criteria. Disagreements regarding eligibility were resolved via discussions with a third experienced reviewer. Reviewers validated questions regarding any study information relevant to eligibility criteria by contacting study authors. Excluded studies were recorded with reason for exclusion. Six reviewers extracted data independently and in duplicate from each study deemed eligible. Reviewers were trained on data extraction protocol prior to extraction. The inclusion and exclusion criteria are presented in Figure 1.

The patient population included CML patients taking the following TKIs: (“imatinib” OR “dasatinib” OR “nilotinib” OR “bosutinib” OR “ponatinib” OR “asciminib” OR “radotinib” OR “ruxolitinib” OR “tipifarnib”). No restriction was placed on participants based on age or phase of CML. In the case of duplicate patient populations identified in multiple peer-reviewed academic articles (such as studies published referencing duplicated NCT numbers), the clinical data for the specific cohort was only recorded once to prevent analytical bias.

Interventions to be considered in this study included the following TKIs: (“imatinib” OR “dasatinib” OR “nilotinib” OR “bosutinib” OR “ponatinib” OR “asciminib” OR “radotinib” OR “ruxolitinib” OR “tipifarnib”. Studies in which treatment did not solely comprise the use of an included TKI were excluded from this analysis.

There were no restrictions for study inclusion based on follow-up time or study. Included articles were reported in the English and Chinese languages. Extracted data was curated from studies published in English. All articles were sourced from Pubmed.gov and Clinicaltrials.gov. Full text journal articles were obtained through open access databases or paid subscriptions through the Georgia Institute of Technology e-journal library.

The primary outcome measure was the quantitative prevalence identified of hematological AEs as a function of TKI type and CML phase. Before curation, the number of unique data points found were: 6 for aplastic anemia, 51 for anemia, 61 for neutropenia, 68 for thrombocytopenia, 58 for myelosuppression, and 18 for pancytopenia. After gathering all found literature through open access or paid subscriptions through the Georgia Institute of Technology e-journal library, studies with one or more of the following attributes were excluded before or during curation: quantitative prevalence data unavailable, published in non-English language, population not comprising diagnosed CML patients, and treatments that did not solely comprise the use of an included TKI. In the case of duplicate patient populations identified in multiple peer-reviewed academic articles (such as studies published referencing duplicated NCT numbers), the clinical data for the specific cohort was only recorded once to prevent analytical bias.

### 2.3. Risk of Bias and Certainty of Evidence

The main objective of the meta-analysis was to objectively assess the existence and prevalence of hematological conditions for CML patients on TKIs. Risk of bias was considered at the study level. In the case of multiple datasets originating from the same clinical study, only the study with the largest listed population size was included in the analysis. This prevented duplicitous inclusion of data from the same clinical dataset. Additionally, bias when comparing TKIs was holistically assessed by examining specific attributes (when provided), including: the TKI median dose, the study median follow-up period, and whether the study included patients who were previously intolerant or resistant to another TKI.

### 2.4. Data Curation and Quality Control

Initially, data recorded from each study included the following: PMID (for paper identification), number of patients under treatment, number of patients under treatment with hematological AEs, fraction of patients with hematological AEs, TKI type [imatinib, dasatinib, nilotinib, bosutinib, ponatinib, asciminib, radotinib, ruxolitinib, tipifarnib], type of hematological condition [aplastic anemia, anemia, neutropenia, thrombocytopenia, myelosuppression, pancytopenia], grade of condition [all grades (grades 1–4) or severe (grade 3–4)], phase of CML [chronic, accelerated, blast], and data source. AE grade, CML phase, TKI dosage and treatment length will only be recorded if available, and otherwise marked as N/A. The absence of these factors did not exclude the study from eligibility. Following initial curation, data was collected and aggregated for “all grades” of the hematological condition. Then, the exact source of each study was traced from the original source, and data published with duplicated NCT numbers were excluded. Lastly, all data curated was independently validated by a quality control team [27,28]. Appendix A illustrates the tabular data collected with corresponding original data source citation.

At the end of the curation and quality control, the number of unique data points collected were as follows: 3 for aplastic anemia, 50 for anemia, 55 for neutropenia, 61 for thrombocytopenia, 13 for myelosuppression, and 16 for pancytopenia. Many studies included data for multiple hematological conditions at the same time, making the number of unique studies different from the number of unique data points. Ultimately, the number of available studies and/or the aggregate sample size of patients for myelosuppression, aplastic anemia, and pancytopenia was too small to be included in the pairwise statistical analysis. Similarly, the number of available studies and/or the aggregate sample size of patients taking ponatinib, asciminib, radotinib, ruxolitinib, or tipifarnib was too small to be included in the pairwise statistical analysis.

### 2.5. Statistical Analysis

Two statistical techniques were used in this meta-analysis: pairwise statistical analysis and odds ratio analysis. Pairwise statistical analyses were performed to examine hematological AE prevalence among CML patients taking TKIs. Pairwise comparisons were performed to examine potential significant differences in hematological AEs as a function of specific TKI therapy. Pairwise comparisons were also performed to examine potential significant differences in hematological AEs as a function of CML phase (chronic, accelerated, and blast phase). The pairwise tests determined if the proportion of patients with the hematological AE was significantly different than the proportion of patients who did not have the hematological AE. The family-wise alpha was set at 0.05. However, the final *p*-value threshold for significance was lowered using a Bonferroni correction to account for multiple comparisons and minimize the likelihood of a Type I error. Additionally, to compare hematological conditions as a function of specific TKI therapy, an odds ratio (OR) with 95% confidence interval was calculated using imatinib as the control population. The objective of performing the OR test was to assess the association of dasatinib, nilotinib, or bosutinib on the odds of getting a specific hematological AE as compared to imatinib. Imatinib was chosen as the control cohort for the OR analysis because it is a first generation TKI and is still viewed as the most popular first-line standard of care worldwide.

## 3. Results

A quantitative statistical meta-analysis was performed to assess the prevalence of the most common hematological conditions in CML patients as a function of hematological AE type, TKI type, and CML disease stage. Appendix A illustrates the aggregated tabular curated data. Imatinib, dasatinib, nilotinib, and bosutinib were the TKI therapies that had sufficient data to be included for pairwise statistical analysis. Anemia, thrombocytopenia, and neutropenia were the hematological conditions that had sufficient data to be included for pairwise statistical comparison.

### 3.1. Assessment of Potential Bias

In any meta-analysis, it is important to assess potential bias based on the attribute properties of included studies. Known biases that could impact results in the assessment of hematological AEs in treatment with TKIs include: CML phase, treatment dose, TKI treatment duration within the study, and inclusion of TKI resistant or intolerance. While this meta-analysis was not able to normalize for all of these attributes, each was holistically considered and quantified where possible.

CML phase was considered when examining the prevalence of hematological adverse events across all TKIs. However, due to sample size, phase was not able to be separated when examining each, individual TKI.

Treatment dose can impact adverse events given higher doses of a TKI is usually thought to increase the likelihood of adverse events. Of the included studies that clearly denoted the dose corresponding to quantitative AE data, the preponderance of studies reported an imatinib dose of 400 mg/day with a range of 200 mg/day to 800 mg/day; a bosutinib dose of 500 mg/day; a dasatinib dose of 140 mg/day with a range of 50–140 mg/day; and a nilotinib dose of 400 mg/day with a range of 200 mg/day to 600 mg/day.

**Figure 2:**
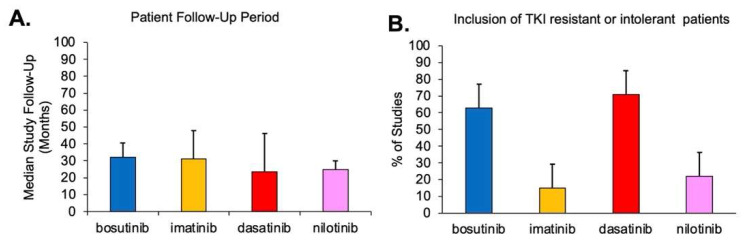
Assessment of study attributes that may impact meta-analysis bias. Not all included studies reported all attributes. Data shown is only for studies that did include a description of attributes. (**A**) The median patient follow-up period (in months) for studies examining bosutinib, imatinib, dasatinib, or nilotinib. Error bars represents interquartile range. (**B**) The percent of included studies examining bosutinib, imatinib, dasatinib, or nilotinib that specifically included patients who had resistance or intolerance to at least one TKI. Error bars represents interquartile range.

Patient follow-up period is important since the majority of adverse events are seen within the first months of starting treatment. Longer follow-up periods are likely to have fewer report adverse events [29]. Not every included study reported the median follow-up period for their reported quantitative data. The aggregate median follow-up period and interquartile range is reported for each TKI. As shown in Figure 2A, imatinib and bosutinib had the longest median follow-up periods of approximately 32 months, while dasatinib and nilotinib had similar but shorter follow-up periods of approximately 24 months.

The inclusion of patients who had previously resistant or intolerant to another TKI can also potentially bias findings as their adverse event profiles could be different than those of previous TKI naïve patients. Not every included study reported whether patients had previously had TKI resistance or intolerance or acquired it during the course of the study. In Figure 2B, it is shown that dasatinib and bosutinib has substantially more studies that included TKI resistant or intolerant patients compared to imatinib or nilotinib.

### 3.2. Assessment of AEs as a Function of CML Phase

**Figure 3:**
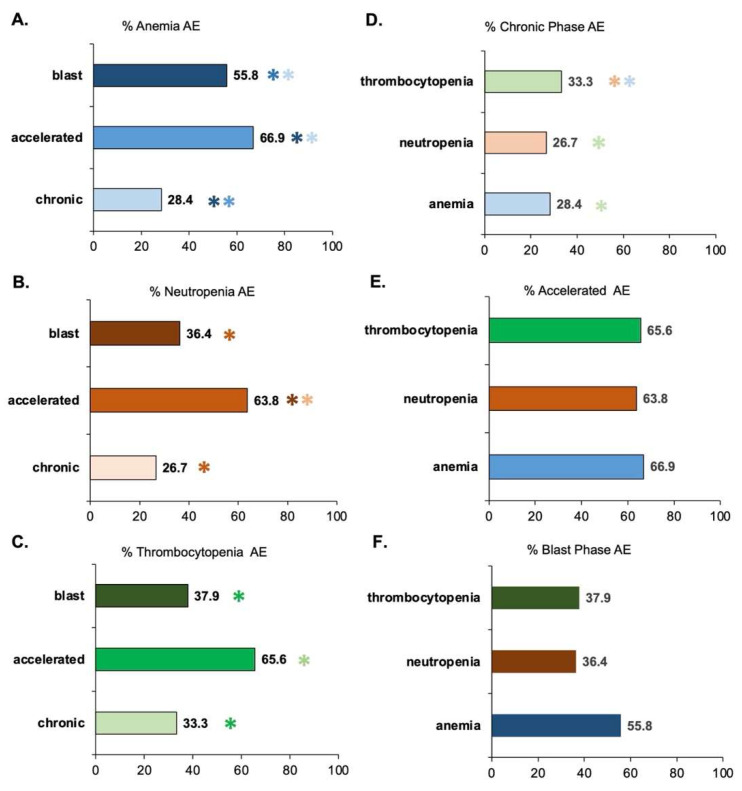
Pairwise statistical comparison hematological AEs (all severities) in CML patients treated with TKIs as a function of CML stage (chronic, accelerated, or blast phase). Percentage is aggregate prevalence of the sample. For pairwise analysis at an alpha of 0.05, significance meeting the Bonferroni-corrected *p*-value of *p* < 0.016 is illustrated with an asterisk (*). The * are colored-coded to illustrate with which other category there is a significant difference. Multiple asterisks (**) of different colors indicate multiple pairs of significant differences. (**A**) Percentage of patients with anemia. There is a significant difference in anemia between every phase pair. (**B**) Percentage of patients (aggregate prevalence) with neutropenia. Neutropenia is significantly different between blast and accelerated phase, as well as chronic and accelerated phase. (**C**) Percentage of patients with thrombocytopenia. Thrombocytopenia is significantly different between blast and accelerated phase, as well as chronic and accelerated phase. (**D**) Percent of chronic phase patients with hematological AE. There is a significant pairwise difference between thrombocytopenia and neutropenia, as well as thrombocytopenia and anemia. (**E**) Percent of accelerated phase patients with hematological AE. There are no statistical differences between any pair of hematological AEs. (**F**) Percent of blast phase patients with hematological AE. There are no statistical differences between any pair of hematological AEs. However, with a larger sample size, there could be significance between anemia and neutropenia or thrombocytopenia.

Statistical analysis was performed to examine significant differences in AE prevalence (all severities) as a function of CML phase (chronic, accelerated, blast) in Figure 3. A pairwise statistical comparison was performed at an alpha of 0.05. Bonferroni correction for multiple comparisons lowered the *p*-value threshold of significance to *p* < 0.016 to avoid a Type I error. Anemia (Figure 3A) has significant differences between each phase: chronic, 28.4%; accelerated, 66.9%; blast, 55.8%. Neutropenia (Figure 3B) had significant differences in prevalence between blast (36.4%) and accelerated phase (63.8%) as well as chronic (26.7%) and accelerated. Thrombocytopenia (Figure 3C) has significant differences in prevalence between blast phase (37.9%) and accelerated (65.6%), as well as chronic phase (33.3%) and accelerated phase.

When comparing differences in hematological AE in all chronic phase patients (Figure 3D), there is a significant difference between anemia (28.4%) and thrombocytopenia (33.3%), as well between thrombocytopenia and neutropenia (26.7%). There were no pairwise significant differences between hematological AE types in accelerated phase patients (Figure 3E): thrombocytopenia (65.6%), neutropenia (63.8%), and anemia (66.9%). Likewise, there were no pairwise significant differences between hematological AE types in blast phase patients: thrombocytopenia (37.9%), neutropenia (36.4%), anemia (55.8%). The *p*-values indicate that anemia is trending towards being significantly more prevalent than neutropenia (*p* = 0.03) or thrombocytopenia (*p* = 0.05). The relationships are shown as insignificant here since they exceed the Bonferroni threshold for significance in multiple comparisons. Nonetheless, with a less conservative post-hoc correction or a larger blast phase patient sample size, anemia (55.8%) may become significantly more prevalent in blast phase patients compared to either thrombocytopenia (37.9%) or neutropenia (36.4%).

### 3.3. Assessment of AEs as a Function of TKI Therapy

**Figure 4:**
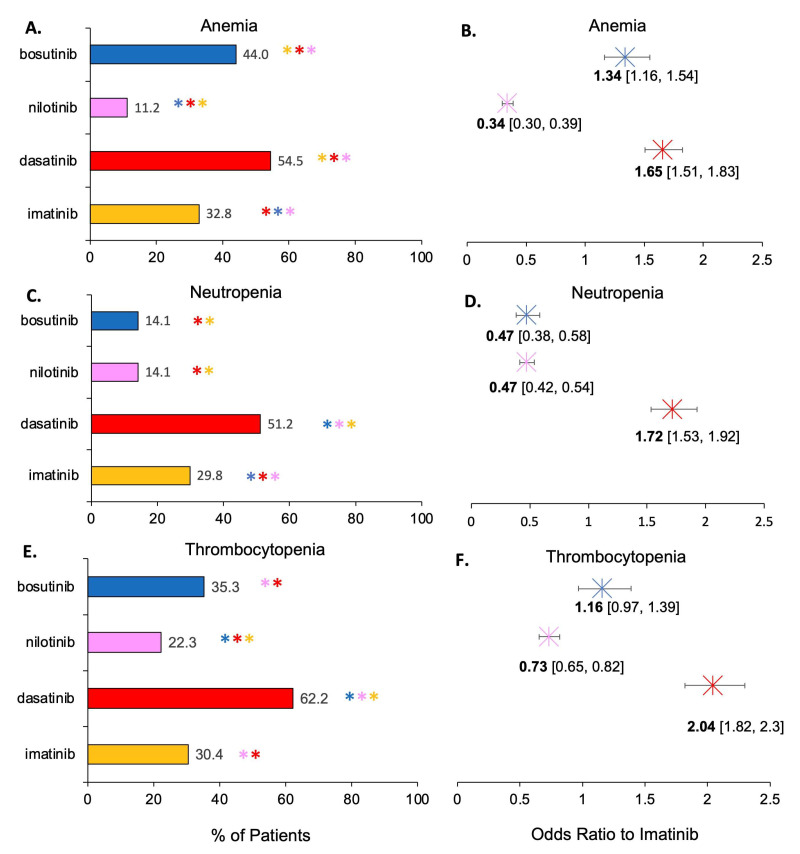
Statistical comparison of hematological AEs (all severities, all CML phases) in CML patients treated with different TKIs. Percentage is aggregate prevalence of the sample. For pairwise analysis at an alpha of 0.05 (Panels **A**,**C**,**E**), significance meeting the Bonferroni-corrected *p*-value of *p* < 0.005 is illustrated with an asterisk (*). The * are colored-coded to illustrate with which other TKI there is a significant difference. Multiple asterisks (**) of different colors indicate multiple pairs of significant differences. For odds ratio analysis (Panels **B**,**D**,**F**), odds of getting the hematological AE are calculated relative to imatinib. Error bars illustrate the 95% confidence interval (CI) and the exact CI is shown inside brackets. An OR > 1 illustrates increased odds of AE compared to imatinib, and an OR < 1 illustrates decreased odds compared to imatinib. (**A**). Pairwise analysis of percentage of patients with anemia based on TKI therapy usage. (**B**). Odds ratio of anemia for each TKI compared to imatinib. (**C**). Pairwise analysis of percentage of patients with neutropenia based on TKI therapy usage. (**D**). Odds ratio of neutropenia for each TKI compared to imatinib. (**E**). Pairwise analysis of percentage of patients with thrombocytopenia based on TKI therapy usage. (**F**). Odds ratio of thrombocytopenia for each TKI compared to imatinib.

Next, statistical analysis was performed to examine significant differences in AE prevalence (all severities) as a function of TKI therapy in Figure 4. A pairwise statistical comparison was performed at an alpha of 0.05. Bonferroni correction for multiple comparisons lowered the *p*-value threshold of significance to *p* < 0.005 to avoid a Type I error. Additionally, an odds ratio with 95% confidence interval was calculated to determine whether a TKI increased or decreased the odds of hematological AE compared to imatinib. Imatinib was used as the control in the odds ratio analysis since it is the oldest and most popular standard of care treatment. Figure 4A–C illustrates pairwise statistical significance analysis between drugs for each hematological condition. Figure 4D–F illustrates the corresponding odds ratio analysis for each TKI and hematological condition.

Prevalence of anemia (all severities, all CML phases) was significantly different between every TKI therapy (Figure 4A). The odds ratio analysis for anemia (Figure 4B) agreed with the pairwise analysis. The odds ratios (OR) and their 95% confidence intervals exceed one for bosutinib (OR = 1.34) and dasatinib (OR = 1.65), meaning both had significantly increased odds of anemia over imatinib. In contrast, nilotinib (OR = 0.34) had significantly decreased odds of anemia compared to imatinib. The percentage of patients with neutropenia (all severities, all CML phases) was significantly higher between dasatinib and all other drugs while being significantly lower between imatinib and all other drugs (Figure 4C). The odds ratio analysis agrees for neutropenia (Figure 4D) as well as the pairwise analysis. The OR and corresponding confidence interval was greater than one for dasatinib (OR = 1.72), meaning dasatinib significantly increased the odds of neutropenia over imatinib. In contrast, bosutinib (OR = 0.47) and nilotinib (OR = 0.47) had significantly decreased odds of neutropenia compared to imatinib. The percentage of patients with thrombocytopenia was significantly different in every pairwise comparison except bosutinib and dasatinib (Figure 4E). The OR and corresponding confidence intervals for thrombocytopenia (Figure 4F) illustrated no significant difference between bosutinib and imatinib. While the bosutinib odds ratio for thrombocytopenia was 1.16, its 95% confidence interval [0.97, 1.39] crossed one. Thus, there was no significant difference in odds of getting thrombocytopenia with bosutinib compared to imatinib. Nilotinib (OR = 0.73) had decreased odds of thrombocytopenia compared to imatinib. Dasatinib (OR = 2.04) had significantly increased odds of thrombocytopenia compared to imatinib.

**Figure 5:**
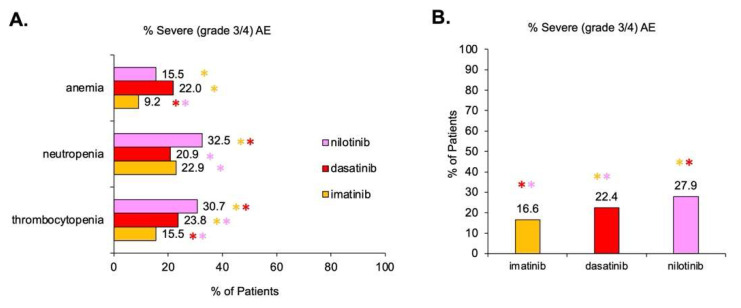
Statistical comparison of severe (grade 3 or 4) hematological AEs (all severities, all CML phases) in CML patients treated with different TKIs. Percentage is aggregate prevalence of the sample. Significance meeting the Bonferroni-corrected *p*-value is shown with an asterisk (*). The * are colored-coded to illustrate with which other TKI there is a significant difference. Multiple asterisks (**) of different colors indicate multiple pairs of significant differences. There was insufficient data for bosutinib. (**A**) Percent of severe hematological AEs separated by TKI type (imatinib, dasatinib, nilotinib). AE type (anemia, neutropenia, thrombocytopenia). (**B**) Aggregated percent of severe hematological AEs for each TKI.

In Figure 5, severe hematological AEs (grade 3 or 4) were compared on the basis of TKI utilizing data presented in Appendix A. Notably, there was only sufficient data to compare imatinib, dasatinib, and nilotinib. CML patients were aggregated across all phases. Dasatinib had a significantly (*p* < 0.05) higher prevalence of severe (grade 3 or 4) anemia (22%) compared to imatinib (9.2%). The prevalence of severe anemia between dasatinib and nilotinib (15.5%) was insignificant (*p* > 0.05) with Bonferroni correction; however, the borderline *p*-value of 0.054 suggests that a larger sample size would likely trend towards being significantly different. The prevalence of severe neutropenia was significantly greater with nilotinib (32.5%) compared to dasatinib (20.9%) and imatinib (22.9%); however, there was no difference (*p* > 0.05) between dasatinib and imatinib. The prevalence of severe thrombocytopenia was significantly greater (*p* < 0.05) with nilotinib (30.7%) compared to dasatinib (23.8%) and imatinib (15.5%). The overall prevalence (aggregated across all AE types) was significantly different between all TKIs (*p* < 0.05) for nilotinib (27.9%), dasatinib (22.4%), and imatinib (16.6%) as shown in Figure 5B.

## 4. Discussion

Previous research incorporating the use of innovative text mining analyses with machine learning illustrated that myelosuppressive hematological conditions (namely anemia, neutropenia, thrombocytopenia, and pancytopenia) were the most connected to TKI usage for CML [13]. The presented results indicated that at least 33% of all chronic phase CML patients actively taking TKIs, whether for active chronic phase CML or in deep remission, presented with anemia, neutropenia, or thrombocytopenia. The results of this meta-analysis illustrate an association between TKI therapy and hematological AEs. Hematological AEs may accompany CML or have a physiological cause. However, the large percentage of AEs among patients on long-term treatment, many which reach deep molecular remission, suggests the need for careful, personalized selection of first-line TKI treatment type and careful TKI dose monitoring to minimize AEs.

### 4.1. Some TKIs Have a Greater Association with Hematological AEs

Patients taking dasatinib had the highest overall prevalence of hematological AEs. The lowest overall prevalence of hematological AE’s occurred for patients on nilotinib. However, when examining only severe (grade or 3 or 4) hematological AEs, nilotinib had the highest overall prevalence and imatinib the lowest overall prevalence. Across each category, dasatinib was associated with a significantly greater prevalence of anemia (including all grades as well as only severe grade 3 or 4 anemia) compared to the other evaluated TKIs.

These findings are corroborated by a recent work [12], which classified dasatinib as the least safe of the 5 FDA approved TKIs (imatinib, dasatinib, nilotinib, ponatinib, bosutinib). Despite an FDA black box warning for Q-T prolongation, this same study found that the safest TKI was nilotinib. Nilotinib had SUCRA (surface under cumulative ranking curve) values consistently 50% lower than those of dasatinib for all four hematological conditions evaluated (anemia, leucopenia, neutropenia, and thrombocytopenia). It was suggested that a selective binding property of nilotinib may contribute to the contrast in safety profile with drugs like dasatinib. Notably, the 2022 meta-analysis excluded both accelerated and blast phases of CML [12].

Delineation between hematological conditions due to TKI usage and those due to CML, itself, can be seen in Figure 4D–F, which visualized the conditions’ prevalence separated by CML phase. Anemia is expected to be common during blast and accelerated phase CML [30], but less common in adult chronic phase CML. Patients in a deep molecular response would not be expected to have any hematological AEs attributed to CML, itself. The development of myelofibrosis associated with over-suppression of bone marrow from imatinib usage has been documented in literature [31]. TKI-related myelofibrosis is a type of secondary myelofibrosis that causes scarring in the bone marrow, which results in decreased production of RBCs and platelets.

### 4.2. The Role of Dose Titration to Minimize Toxicity and AEs

Different TKIs have varying pharmacokinetics and chemical structures that impact their absorption and likelihood to cause specific AEs, including the prevalence of hematological AEs [32]. Titration of TKI dosage can be implemented to prevent conditions associated with TKI usage, such as those stemming from severe bone marrow suppression or myelofibrosis. TKI dose lowering has been tried in multiple studies in order to minimize AEs while still maintaining a sufficient suppression of BCR-ABL leukemic cell line [33]. In particular, dose lowering is often tried in second generation TKIs to reduce toxicities. A dose lowering from 100 mg/day to 50 mg/day of dasatinib was found to be well tolerated in newly diagnosed chronic phase CML patients [34]. The DESTINY trial examined dose de-escalation in patients on imatinib, dasatinib, or nilotinib and found that dose de-escalation was safe with only 7% of patients having a molecular recurrence after 12-months [35]. Besides dose titration, other regimes have tried alternating periods of taking a TKI with periods of not taking a TKI in order to reduce toxicity and AEs. An Italian study examining alternating 1-month periods of imatinib treatment in elderly patients found that about 17% of patients lost a cytogenetic response with a follow-up for 4-years [36]. These are a few examples of studies that have shown that careful selection of patients to receive lower doses of TKIs may be a viable option to reduce toxicities and AEs. In particular, dose lowering may be a beneficial strategy in patients who are not otherwise successful in fully stopping TKI therapy due to either a failed TFR or clinical ineligibility to attempt a TFR.

### 4.3. Personalized TKI Selection to Minize AEs

Many factors must be considered when selecting the optimal TKI for a given CML patient [37]. Literature has documented a correlation between age and anemia prevalence at CML diagnosis. Furthermore, anemia prevalence at diagnosis was determined as a prognostic factor for evaluating deep molecular response [8]. Delays in achieving major and deep molecular responses were significantly increased for pediatric CML patients with moderate and severe anemia; also patients with anemia were more likely to fail on imatinib treatment [8]. In addition, TKIs have been shown to exacerbate other non-hematological co-morbidities, such as iron homeostasis and thyroid function [13]. Iron-deficiency induced anemia has been associated with decreased thyroid functioning, which provides insight into the interconnections between TKIs and both hematological and non-hematological comorbidities. Machine learning of text relationships that TKIs are reducing absorption of nutrients like iron, which increases the likelihood of anemia [13].

The results of this and related studies suggest that a deep molecular response does not preclude the occurrence or reoccurrence of hematological AEs later in therapy and during remission periods. Prevalence of hematological AEs associated with TKI usage indicates a need for regular patient monitoring, which should include at a minimum peripheral complete blood count tests. Known co-morbid conditions or AEs that are initiated or exacerbated by TKIs that contribute to hematological AEs include iron deficiency, thyroid dysfunction, and kidney dysfunction [13]. Physicians and healthcare providers can utilize information regarding AE prevalence for each TKI type to tailor a specific treatment plan based on patient history and genetic predisposition. Previous work has characterized the prevalence of gastrointestinal AEs related to TKI type in the treatment of CML [28]. Moreover, cross-domain text mining predicted less common or even novel adverse events which are not fully understood [13]. The use of predictive analysis and algorithms in conjunction with clinical evidence can provide a basis for an individualized treatment plan.

### 4.4. Investigation Limitations and Future Directions

The primary limitation of the present study was presented in the assessment of potential biases in Figure 2. Specifically, due to sample size limitations, the study treatment dose, treatment follow-up duration, inclusion of resistant or intolerant patients was not factored into the quantitative aggregate meta-analysis results. Figure 2 illustrated that, of the included studies that reported it, the median follow-up period for imatinib and bosutinib studies was larger than that of dasatinib and nilotinib. Additionally, dasatinib, which was the first TKI to be released after imatinib, has more studies that contained patients who were either resistant or intolerant to imatinib. Treatment dose is also a consideration, although the median treatment dose for the preponderance of included studies was similar for a given TKI. Collectively, the bias analysis and data presented in Figure 2 illustrated that there is more potential bias among the dasatinib studies compared to the other TKIs. As such, this bias could potentially over-represent hematological AEs for dasatinib in this meta-analysis. However, it is also unlikely that this bias solely accounted for the significantly disproportionate number of hematological AEs with dasatinib, especially anemia, compared to the other TKIs examined in this meta-analysis.

Regardless of TKI type, patients earlier in their TKI treatment or on higher doses of TKI means are known to have more adverse events. The number of hematological AEs earlier in treatment is greater due to efficacy of TKIs in eliminating the clonal cell line. After the leukemic cells are eradicated, the cytopenias lessen. In this meta-analysis, imatinib had the largest range of treatment follow-up (a median of 31.1 months ± 16.1 months), which is attributed to it being the first broadly used TKI and a common comparator group for more newly released TKIs. In contrast, one of the studies included in this meta-analysis, a single large cohort study by Kalmanti and colleagues [38] reported hematological AEs with imatinib over a 10-year (120-month) follow-up period. The prevalence of hematological AEs in patients treated over 120-months in their study [38] was about one-third lower than the aggregate prevalence of hematological AEs in the present meta-analysis.

As more long-term and dose titration studies become available, future work will become possible that simultaneously examines the impact of resistance/intolerance, treatment dose, treatment duration, etc. Such work will help improve personalized TKI selection and optimal patient monitoring.

## 5. Conclusions

CML is a relatively rare condition and TKIs are still relatively new, with only about 20 years since the first TKI, imatinib, came to market. Approximately 1 of 3 TKI patients experienced a hematological AE while on TKI therapy. Moreover, there were significant differences in hematological AE prevalence between TKI drug types. There were variations in prevalence based on specific AE type (anemia, neutropenia, thrombocytopenia). Nonetheless, the overall prevalence of hematological AEs by TKI type was: dasatinib > bosutinib > imatinib > nilotinib. Dasatinib consistently had the highest prevalence of anemia for all grades and specifically for severe grades 3 and 4. When specifically examining only severe AEs, nilotinib had the highest overall prevalence and imatinib the lowest overall prevalence of grade 3 or 4 hematological AEs. Personalized TKI selection based on individualized AE risk and comorbidities may reduce the incidence of AEs occurring in patients on long-term TKI therapy.

## Data Availability

All data utilized is provided in Appendix A.

## Appendix A

**Table A1:** A complete list of the data acquired during the literature-based meta-analysis to evaluate prevalence of hematological AEs (anemia, neutropenia, thrombocytopenia) as a function of type of tyrosine kinase inhibitor (TKI), AE severity, and CML disease stage (chronic, accelerated, blast). In the table below, N/A stands for not applicable or no applicable data source available. Example calculation: To calculate the % of patients with anemia for all grades, imatinib, (#WithCondition)/(Total#) = 1036/(1036 + 1738) = 37.3%. Reference number corresponds to the references in the main manuscript reference list. Note that some patients have more than one adverse event, which makes the total percentage of all hematological AEs combined appear to be greater than 100%.

Hematological Condition	TKI Type	# of Patients with Condition from Studies of: (All Grades | Grade 3–4 Only)		# of Patients without Condition from Studies of: (All Grades | Grade 3–4 Only)		% of Patients with Condition from Studies of: (All Grades | Grade 3–4 Only)		Reference Number
Anemia	Imatinib	1175	76	2404	1419	32.8	9.17	[38,39,40,41,42,43,44,45,46,47,48,49,50,51,52,53,54,55,56]
	Dasatinib	1244	121	1040	430	54.4	22	[47,56,57,58,59,60,61,62,63,64,65]
	Nilotinib	299	71	2376	387	11.2	15.5	[66,67,68,69,70]
	Bosutinib	343	N/A	437	N/A	44	N/A	[54,55,71]
	Ponatinib	105	N/A	312	N/A	25.2	N/A	[72]
	Asciminib	17	N/A	133	N/A	11.3	N/A	[73]
	Radotinib	48	4	112	73	30	5.2	[20,50]
	Ruxolitinib	24	N/A	36	N/A	40	N/A	[74,75]
	Tipifarnib	17	N/A	9	N/A	65.4	N/A	[22]
Neutropenia	Imatinib	1004	274	2365	1536	29.8	22.89	[38,39,40,41,43,44,45,46,47,48,49,50,51,52,53,54,56,76,77,78,79]
	Dasatinib	736	235	702	890	51.2	20.9	[47,58,59,60,61,62,63,64,65,80,81]
	Nilotinib	362	251	2206	521	14.1	32.5	[66,67,68,70,82,83,84]
	Bosutinib	110	N/A	670	N/A	14.1	N/A	[54,71,76]
	Ponatinib	126	N/A	480	N/A	20.8	N/A	[72,79,85]
	Asciminib	16	N/A	134	N/A	10.7	N/A	[73]
	Radotinib	61	1	99	76	38.1	1.3	[20,50]
	Ruxolitinib	N/A	N/A	N/A	N/A	N/A	N/A	N/A
	Tipifarnib	12	N/A	14	N/A	46.2	N/A	[22]
Thrombocytopenia	Imatinib	1075	308	2363	1854	30.4	15.5	[38,39,40,41,43,44,45,46,47,48,49,50,51,52,53,54,55,56,77,78,79,86,87,88]
	Dasatinib	706	365	429	1171	62.2	23.8	[47,58,59,60,61,62,63,64,65,80,81,88,89]
	Nilotinib	601	237	2095	535	22.3	30.7	[66,67,68,69,70,82,84]
	Bosutinib	188	N/A	344	N/A	35.3	N/A	[54,55,71]
	Ponatinib	262	N/A	344	N/A	43.2	N/A	[72,79,85]
	Asciminib	33	N/A	117	N/A	22	N/A	[73]
	Radotinib	142	N/A	95	N/A	59.9	N/A	[20,50]
	Ruxolitinib	N/A	N/A	N/A	N/A	N/A	N/A	N/A
	Tipifarnib	10	N/A	16	N/A	38.5	N/A	[22]
